# Motor and sensory features successfully decode autism spectrum disorder and combine with the original RDoC framework to boost diagnostic classification

**DOI:** 10.1038/s41598-021-87455-w

**Published:** 2021-04-09

**Authors:** Laura A. Harrison, Anastasiya Kats, Emily Kilroy, Christiana Butera, Aditya Jayashankar, Umit Keles, Lisa Aziz-Zadeh

**Affiliations:** 1 USC Chan Division of Occupational Science and Occupational Therapy, University of Southern California, Los Angeles, CA USA; 2 Brain and Creativity Institute, University of Southern California, Los Angeles, CA USA; 3 Division of Humanities and Social Sciences, California Institute of Technology, Pasadena, CA USA

**Keywords:** Autism spectrum disorders, Developmental disorders, Cognitive neuroscience, Diseases of the nervous system, Motor control, Sensorimotor processing, Sensory processing, Social behaviour, Social neuroscience, Neurodevelopmental disorders, Autism spectrum disorders, Biomarkers, Comorbidities, Neuroscience, Psychology, Human behaviour

## Abstract

Sensory processing and motor coordination atypicalities are not commonly identified as primary characteristics of autism spectrum disorder (ASD), nor are they well captured in the NIMH’s original Research Domain Criteria (RDoC) framework. Here, motor and sensory features performed similarly to RDoC features in support vector classification of 30 ASD youth against 33 typically developing controls. Combining sensory with RDoC features boosted classification performance, achieving a Matthews Correlation Coefficient (MCC) of 0.949 and balanced accuracy (BAcc) of 0.971 (*p* = 0.00020, calculated against a permuted null distribution). Sensory features alone successfully classified ASD (MCC = 0.565, BAcc = 0.773, *p* = 0.0222) against a clinically relevant control group of 26 youth with Developmental Coordination Disorder (DCD) and were in fact required to decode against DCD above chance. These findings highlight the importance of sensory and motor features to the ASD phenotype and their relevance to the RDoC framework.

## Introduction

The Research Domain Criteria (RDoC) is a framework developed by the National Institute of Mental Health (NIMH) that aims to describe functioning across several psychological domains and neurobiological levels of analysis^1^. The domains and techniques specified by the RDoC matrix reflect current research priorities and may influence the scope of planned research programs. The original RDoC matrix included five domains: (1) negative and (2) positive valence; (3) cognitive systems; (4) social processes; and (5) arousal and regulatory systems. In January 2019, a sixth *sensorimotor systems* domain focusing on motor control and learning was added^2^. In the same month, we proposed the further addition of a domain focused on sensory and perceptual processing due to the prevalence of sensory processing atypicalities in several mental health disorders^3^.

The objective of the current paper is to provide a testbed for exploring the supposition that adding motor and sensory domains to the original RDoC domains better captures heterogeneity within a mental health disorder. We also aim to test the relative descriptive success of these domains separately (motor; sensory; original RDoC). We explore these questions with the ASD phenotype, a developmental disorder that persists throughout life and is notable for the diversity of its presentation and clinical outcomes across individuals. As such, ASD is an apt target for investigating the interplay of these various neurobehavioral domains. We tested the capability of the RDoC to capture the ASD phenotype in relation to typically developing (TD) controls, and to another clinical control group: developmental coordination disorder (DCD). First, we briefly review what is known about these two clinical disorders with respect to the original RDoC domains as well as with respect to sensory and motor functioning.

### Autism spectrum disorder (ASD)

#### Definition and diagnosis

ASD affects 1 in 59 children in the United States and impacts four times as many boys as girls^4^. Current diagnostic criteria from the DSM-5 include (1) social-communication differences and (2) restricted, repetitive behavior (RRB).

#### RDoC domains and ASD

Atypicalities associated with ASD span all current RDoC domains. Regarding the positive valence system domain, aberrant reward circuits, especially regarding social rewards and motivation, may underlie ASD symptomatology^5,6^. Considering the negative valence systems domain, approximately 40% of individuals with ASD have at least one co-occurring anxiety disorder^7,8^. Estimates of increased non-clinical anxiety-related behaviors in children with ASD are broad, ranging from 11 to 84%, with discrepancies attributable to differences in the samples studied and assessments employed^8^.

Within the cognitive systems domain, executive dysfunction has been suggested to account for symptoms of inflexibility, lack of inhibition, and difficulty with metacognitive self-monitoring in ASD. A recent meta-analysis indicates a small to medium effect of executive function in ASD^9^. Additionally, approximately 22–83% of individuals with ASD also meet the DSM-IV criteria for Attention Deficit and Hyperactivity Disorder (ADHD)^10^.

Social and communication differences are core symptoms of ASD and reflect the social processes domain—including (1) affiliation and attachment, (2) facial and non-facial social communication, (3) self perception, and (4) perception of others, including animacy and action perception and understanding of mental states—have been observed. Finally, abnormal arousal modulation has been observed during direct gaze^11^ and social play^12^ in ASD.

#### Sensory differences in ASD

As sensory processing differences affect 40–90% of children with ASD^13^, the DSM-5 includes sensory sensitivity as a symptom of ASD, and it has been proposed that sensory symptoms are central to the neurobiology of autism^14^. Recent research hypothesizes that sensory differences are correlated with and may even exacerbate symptoms associated with ASD, including social communication^15^, attention^16^, and alexithymia^17^.

Recent research efforts have focused on the neurobiological basis for sensory sensitivity in ASD. Evoked responses in visual, auditory, and somatosensory cortices show more response variability, but not mean amplitude differences in adults with autism relative to controls^18^. Relevant to differences in sensory sensitivity, children with ASD showed increased BOLD contrast to mildly aversive auditory and tactile stimuli in primary sensory cortices and the amygdala even after controlling for anxiety compared to the controls^19^. Additionally, in the same study, connectivity between the right amygdala and orbitofrontal cortex was affected by ASD and sensory sensitivity: connectivity between these regions was slightly positive in the TD control group, while the ASD subgroup without Sensory Over Responsivity (SOR) had the most strongly negative connectivity, and the ASD subgroup with SOR had less negative connectivity^19^. Thus, unlike individuals without ASD or SOR, individuals with ASD and SOR may not use prefrontal regions to down-regulate amygdala activity during sensory experiences.

#### Motor differences in ASD

Alongside sensory differences, motor differences were noted in Kanner’s original report^20^. Individuals with ASD commonly present motor dysfunctions and motor features are beginning to be used by researchers to classify ASD and show clinical promise as a screening tool—in one study it was even possible to classify ASD vs. TD children with 93% accuracy using motor measures from an electronic tablet task^21^. Related to such measurable motor differences, attempts to apply machine learning and machine vision to the automated classification of autism from abnormal movements represent a growing field worthy of separate review^22–24^. It is estimated that 74–100% of individuals with ASD have gross motor impairments^25^. General movement impairments as measured by the Movement Assessment Battery for Children (MABC), were found in 79% of children with ASD, while a further 10% exhibited borderline motor impairments; motor impairments were especially pronounced in ASD children with low-IQ^26^. Furthermore, a meta-analysis indicated that motor impairments are a cardinal feature of ASD^27^. Motor disruption may arise very early in development and contribute to an ASD-specific neurodevelopmental trajectory^28^. With such high rates of motor impairments, DCD is a common co-occurrence^29^, with 79% of children with ASD in a mixed sample including children across the spectrum exhibiting definite movement impairments consistent with DCD, as measured by the MABC, and a further 10% showing borderline impairments^26^. However, there may be subtle differences between the types of motor disturbances individuals with ASD and individuals with DCD portray. For instance, Paquet and researchers^30^ compared several aspects of neuro-psychomotor functioning in 18 children with ASD and two subtypes of DCD; such detailed characterization of motor impairments may be key for differentiating groups.

### Developmental coordination disorder (DCD)

#### Definition and diagnosis

DCD, also known as dyspraxia, is a neuromotor disorder that affects an estimated 5–6% of children^29,31,32^, with some estimating prevalence as low as 2% and as high as 20%^29^. Like ASD, DCD is a heterogeneous condition that may include impairments in fine and/or gross motor skills. DSM-5 criteria for diagnosis include impairments in the development of motor skills and/or coordination to the extent that it interferes significantly with social and academic activities. Additionally, symptoms must be present during the early development period and not explained by other neurological conditions that affect movement, visual impairment, or intellectual delay.

#### Co-occurrence with and distinction from ASD

While a large percentage of children with ASD have co-occurring DCD, about 90% of children with DCD do not have ASD^33^ and the behavioral profiles of each were unique in a meta analysis^34^. One population-based study of 344 children with DCD found 4.1% of children with moderate DCD and 8.2% with severe DCD to have co-occurrence with ASD^33^. Additionally, individuals with DCD may experience subclinical social difficulties^35–37^ as a secondary feature to their primary motor difficulties.

#### RDoC domains and DCD

DCD is not as well studied as ASD, and further research will need to be done to understand the range of ability in the former across the RDoC domains. For example, our review did not find much literature regarding the positive valence systems or arousal/regulatory systems domain in DCD. In relation to the negative valence system domain, one study found that a quarter of individuals with DCD exhibited clinically significant levels of anxiety^38^.

As related to the cognitive systems domain, up to 50% of individuals with DCD present increased impulsivity and decreased attention^29,39^ and DCD often co-occurs with other developmental conditions, including ADHD and dyslexia^32,33^. Reduced cognitive skills related to four stages of information processing from the Das-Naglieri Planning, Attention, Simultaneous and Successive (PASS) theory of cognitive processing have also been observed in DCD^40^. Others have shown that cognitive differences in DCD persist through development and may even be present in adulthood^29^.

Regarding the social processes domain, while social differences in DCD are subclinical and distinct in both quality and severity from those observed in ASD, they are nevertheless regularly observed^29^. Proper motor skills enable individuals to interact with their environment, and movement difficulties seen in individuals with DCD may impede key opportunities for social skill development. Indeed, motor problems in children with DCD have been positively correlated with peer, internalizing, and externalizing difficulties^41^. However, another study found that social difficulties in DCD were not a function of empathic ability, and proposed that they were the result of an accumulation of external factors^42^. Most researchers consider social differences in DCD to be secondary symptoms with poor motor skills resulting in a pattern of repeated feelings of social inadequacy in motor contexts, such as always being chosen last for team sports.

#### Sensory differences in DCD

As motor movement is inherently linked to sensation, differences in one may impact functioning in the other. In particular, individuals with DCD exhibit visual-motor differences. For instance, children with DCD performed significantly worse than controls on the Beery-Buktenica Developmental Test of Visual Motor Integration^43^. Additionally, diffusion magnetic resonance imaging-based tractography showed decreased fractional anisotropy and elevated radial diffusivity (RD) of the left and right retrolenticular limb of the internal capsule in children with DCD compared to controls^43^. Such differences in sensorimotor tracts will need to be further studied to better understand the etiology of sensory differences.

#### Motor differences in DCD

By definition, individuals with DCD exhibit a range of behavioral motor impairments (e.g., balance, fine motor skills, gross motor skills, coordination, etc). Several fMRI studies have investigated the neurobiological basis of these differences, with several motor brain regions showing dysfunctional activity. For instance, during a visuomotor tracking task, individuals with DCD showed less control during the task as well as less activational in the left posterior parietal cortex and left postcentral gyrus as compared to healthy controls^44^. In a trail-tracing task, individuals with DCD had greater activation of the left inferior parietal lobule, right middle frontal gyrus, right supramarginal gyrus, right precentral gyrus, right superior temporal gyrus, and right cerebellar lobule VI compared to controls^45^. Thus, depending on the motor task employed, several motor related brain regions may be activated differently in individuals with DCD compared to TD peers.

### Experimental aims and hypotheses

Distinguishing between disorders that share behavioral features or which have high rates of co-occurrence is clinically challenging, especially when considering disorders like ASD, where—by definition—individual presentations span a spectrum. The RDoC framework aims to circumvent this concern. However, an accurate mapping between diagnostic labels and treatment options is presently still clinically desirable. Ideally, the RDoC framework would not only describe behavior across domains within a disorder (i.e., distinguish a clinical from a healthy control group), but it would also be able to differentiate clinically similar (yet dissociable) diagnoses.

To that end, to assess the relative contribution of (1) behaviors from the original RDoC framework (hereafter, “RDoC”) and (2) sensory and, (3) motor behaviors, which are just now being recognized as important to the RDoC approach^2,3^, in capturing the heterogeneity of the ASD phenotype, we compare the predictive performance of support vector classifiers built using sensory, motor, and RDoC features separately as well as combinations thereof in classifying individuals diagnosed with ASD. First, we compare the relative performance of different psychological models (e.g., motor, sensory, RDoC, and combinations) in the classification of individuals with ASD versus TD controls. After testing the importance of sensory and motor features in capturing the autism phenotype relative to TD controls, we extend this classification to the more difficult, yet more clinically-relevant classification of ASD versus DCD individuals.

Within each classification separately, our primary aim is to compare the performance of motor, sensory, and RDoC features separately—i.e., how does the predictive performance of (1) motor, (2) sensory, and (3) motor and sensory features combined compared to (4) features reflected in the original RDoC matrix? In response to our first aim, our first hypothesis (H1) is that motor and sensory measures, either alone or combined, will perform similarly to RDoC features across decoders (with the exception of motor features alone for the ASD vs. DCD decoder, as we expect substantial overlap in motor characterization between these two groups). Following this hypothesis, we have no specific hypothesis about the relative performance of motor versus sensory features.

Following the primary aim of comparing motor and sensory performance to traditional RDoC performance, we then aim to compare the performance of RDoC features alone to that of the RDoC *combined* with motor and/or sensory measures to determine whether addition of motor and sensory features enhances RDoC-based discrimination. Performance is compared both for complete individual and combined feature sets that have been optimized using feature selection. In relation to this second aim, we hypothesize (H2) that classification performance increases when motor and/or sensory features are added to those reflecting the original RDoC framework.

## Results

### Dataset characterization

Overall, (1) correlations between features sets (Supplementary Figs. S1, S2) showed that RDoC and sensorimotor constructs were non-redundant and (2) paired group comparisons (Tables 1, 2, 3, 4) within feature sets demonstrated that our three subject groups differed on measures across feature categories, with the exception of motor features, for which ASD and DCD subjects scored similarly.

**Table 1:** Importance of motor features in distinguishing ASD.

Feature	ASD vs. TD Cohen’s D	DCD vs. TD Cohen’s D	ASD vs. DCD Cohen’s D	ASD vs. TD selection frequency	ASD vs. DCD selection frequency
MABC total	− 2.38	− 3.45	0.28	NA	NA
MABC manual dexterity	− 1.71	− 2.11	0.14	NA	NA
MABC balance	− 1.53	− 1.96	0.35	NA	NA
MABC aim and catch	− 1.19	− 1.31	− 0.01	NA	NA
DCDQ total	− 3.01	− 2.88	0.13	1	0
DCDQ handwriting	− 2.51	− 2.67	0.20	1	0
DCDQ coordination	− 2.01	− 1.95	0.10	1	0
DCDQ movement	− 1.89	− 2.13	0.23	1	0
DCDQ motor planning	− 1.47	− 0.98	− 0.29	1	0
MABC checklist total	1.62	1.84	− 0.13	1	0
SIPT truescore	− 0.90	− 1.06	− 0.03	0.2	0

The first three columns report Cohen’s *d* effect size from paired comparisons between subject groups. The last two columns report selection frequency of each feature by univariate feature selection for the full RDoC + M + S feature set across all 2000 cross validation loops for each of our two decoders (ASD vs. TD and ASD vs. DCD). Motor features (rows) sorted by selection frequency.

**Table 2:** Importance of sensory features in distinguishing ASD.

Feature	ASD vs. TD Cohen’s D	DCD vs. TD Cohen’s D	ASD vs. DCD Cohen’s D	ASD vs. TD selection frequency	ASD vs. DCD selection frequency
SenSOR food	2.97	1.76	1.33	1	0.955
SenSOR tactile	2.94	1.65	1.36	1	0.9735
SenSOR self care	2.53	1.25	1.08	1	0.4395
SenSOR Smell	2.47	1.58	0.80	1	0.029
SSP behavioral	2.34	0.75	1.42	1	0.986
SenSOR visual	2.12	1.55	0.45	1	0
SSP registration/bystander	1.90	0.71	0.87	1	0.142
SenSOR garment	1.87	1.24	0.78	1	0.0105
SSP sensitivity/sensor	1.64	0.32	1.29	1	0.863
SSP avoiding/avoider	1.64	0.22	1.34	1	0.9165
SenSOR place	1.60	0.39	1.29	1	0.875
SenSOR movement	1.50	0.57	1.06	1	0.294
SenSOR sound	1.38	0.66	0.74	1	0.013
SSP sensory	0.97	0.87	0.00	0.4155	0
SSP seeking/seeker	0.85	0.09	0.81	0.106	0.004
SenSOR total	0.86	0.43	0.45	0.0795	0

The first three columns report Cohen’s *d* effect size from paired comparisons between subject groups. The last two columns report selection frequency of each feature by univariate feature selection for the full RDoC + M + S feature set across all 2000 cross validation loops for each of our two decoders (ASD vs. TD and ASD vs. DCD). Sensory features (rows) sorted by selection frequency.

**Table 3:** Importance of social RDoC features in distinguishing ASD.

Feature	ASD vs. TD Cohen’s D	DCD vs. TD Cohen’s D	ASD vs. DCD Cohen’s D	ASD vs. TD selection frequency	ASD vs. DCD selection frequency
SRS total	3.90	1.56	2.01	NA	NA
SRS social comm. and interaction	3.73	1.48	1.92	NA	NA
SRS social communication	3.52	1.35	1.95	NA	NA
SRS social cognition	2.91	1.06	1.39	NA	NA
SRS awareness	2.70	0.89	1.90	NA	NA
SRS social motivation	2.17	0.93	1.18	NA	NA
SCQ total	1.65	0.58	1.29	1	0.896
CBCL total competence	− 1.61	− 0.48	− 0.98	1	0.2835
CBCL SS social problems	2.00	1.39	0.68	1	0.0045
SCQ recip. social interaction	1.17	0.35	0.89	0.9245	0.057
CBCL SS rule-breaking behavior	0.93	0.77	0.20	0.216	0
SCQ communication	0.83	0.44	0.56	0.097	0
NEPSY ToM total	− 0.75	0.08	− 0.79	0.06	0.0425
NEPSY ToM verbal	− 0.71	0.06	− 0.73	0.0195	0.011
Alexithymia communication	0.46	− 0.08	0.59	0.0005	0.0035
Alexithymia total	0.53	0.15	0.33	0	0
Alexithymia identification	0.38	0.30	0.09	0	0
IRI personal distress	0.34	0.28	0.07	0	0
Alexithymia external thinking	0.23	0.08	0.10	0	0
IRI fantasy scale	0.15	0.16	− 0.02	0	0
EmQue affective	0.13	0.55	− 0.33	0	0
NESPY affect recognition	− 0.45	− 0.18	− 0.23	0	0
LOI how hands	− 0.35	− 0.21	− 0.09	0	0
LOI why hands	− 0.33	− 0.36	0.01	0	0
IRI perspective taking	− 0.32	− 0.13	− 0.21	0	0
LOI why face	− 0.27	− 0.30	0.01	0	0
LOI how face	− 0.24	0.04	− 0.27	0	0
EmQue prosocial motiv	− 0.22	0.12	− 0.33	0	0
IRI empathic concern	− 0.19	0.10	− 0.29	0	0
EmQue cognitive	− 0.17	− 0.04	− 0.11	0	0
NEPSY ToM contextual	− 0.12	0.23	− 0.41	0	0
IRI total	0.00	0.15	− 0.16	0	0

The first three columns report Cohen’s *d* effect size from paired comparisons between subject groups. The last two columns report selection frequency of each feature by univariate feature selection for the full RDoC + M + S feature set across all 2000 cross validation loops for each of our two decoders (ASD vs. TD and ASD vs. DCD). Social features (rows) sorted by selection frequency. In CBCL features, *SS* = syndrome scale.

**Table 4:** Importance of non-social RDoC features in distinguishing ASD.

Feature	ASD vs TD Cohen’s D	DCD vs. TD Cohen’s D	ASD vs. DCD Cohen’s D	ASD vs. TD selection frequency	ASD vs. DCD selection frequency
CBCL SS thought problems	1.77	0.75	1.08	1	0.3885
CBCL SS withdrawn/depressed	1.53	0.65	0.83	1	0.051
Conners ADHD parent report	4.31	2.03	0.74	1	0.018
CBCL SS attention problem	1.80	1.62	0.86	1	0.013
CASI anxiety symptom count	1.21	0.78	0.30	0.9135	0
CBCL SS anxious/depressed	0.98	0.16	0.72	0.4055	0.0285
Conners ADHD child report	0.98	0.66	0.15	0.3735	0
CBCL SS aggressive behavior	0.94	0.61	0.61	0.2875	0
CBCL SS somatic complaints	0.88	0.41	0.55	0.1485	0
WASI-II VIQ	− 0.64	− 0.05	− 0.52	0.0235	0.0005
PANAS negative	0.67	0.45	0.11	0.0085	0
PH-C total arousal	0.66	0.54	0.21	0.0065	0
PANAS positive	− 0.50	− 0.26	− 0.21	0.001	0
WASI-II FSIQ-2	− 0.48	− 0.15	− 0.29	0	0
WASI-II FSIQ-4	− 0.36	− 0.24	− 0.12	0	0
WASI-II PRI	0.02	− 0.31	0.27	0	0

The first three columns report Cohen’s *d* effect size from paired comparisons between subject groups. The last two columns report selection frequency of each feature by univariate feature selection for the full RDoC + M + S feature set across all 2000 cross validation loops for each of our two decoders (ASD vs. TD and ASD vs. DCD). Features (rows) sorted by selection frequency. Features represent the cognitive, arousal, positive and negative valence domains from the RDoC.

Spearman correlations between RDoC and both motor and sensory features (SM Figs. S1, S2) showed that of 42 RDoC features, 9 (21.4%) correlated (*p* < 0.001, uncorrected) with more than one motor feature and 15 (35.7%) correlated with more than one sensory feature, demonstrating that sensory and motor features were non-redundant with RDoC features.

Regarding paired group comparisons, generally, the ASD and TD groups differed the most across measures from all domains, while the DCD group showed intermediate differences from the ASD group, with the smallest differences seen for motor measures. Specific group differences by feature category are detailed below. Absolute Cohen’s *d* cutoffs for descriptive effect sizes were as follows: 0.2 constituted a small effect; 0.5 a medium effect, and 0.8 a large effect.

#### Motor features

All motor features (Table 1) showed a strong effect between TD and ASD subjects, with Cohen’s *d* ranging between 0.90 (SIPT truescore) and 3.01 (DCDQ total). Concordantly, all motor features had univariate feature selection frequency counts greater than our 5% support vector classifier (SVC) inclusion threshold. By definition, the DCD group had greater motor difficulties than the TD group. Effect sizes between DCD and TD subjects were large for all motor features, with Cohen’s *d* ranging between 0.98 (DCDQ motor planning) and 3.45 (MABC total).

Meanwhile, group differences were nonexistent to weak between DCD and ASD subjects, both of whom demonstrated significant motor difficulties relative to TD subjects, with Cohen’s *d* effect sizes ranging between 0.01 (MABC Aim and Catch) and 0.35 (MABC balance). Five motor measures (DCDQ Movement, Motor Planning, and Handwriting, as well as MABC balance and total) showed small effects between the ASD and DCD groups (Table 1). The remaining six motor measures did not differ between the groups. No motor features were ever selected by univariate feature selection for the ASD vs. DCD decoder; therefore, as described in the methods, the motor feature with the greatest difference between these groups outside of the MABC—which was part of our inclusionary criteria and therefore not used in decoding—was the DCDQ Motor planning feature (*d* =  − 0.29), which was hand selected for our ASD vs. DCD motor SVC.

#### Sensory features

All Cohen’s *d* scores indicated large effects between ASD and TD subjects on sensory features (Table 2), with scores ranging between 0.86 (SenSOR total) and 2.97 (SenSOR food); all sensory features were selected by univariate feature selection for ASD vs. TD decoding with a frequency greater than our 5% cutoff (minimum frequency = 0.0795 for SenSOR total).

Differences between TD and DCD subjects were intermediate to and smaller those observed between TD and ASD subjects (see Table 2): 7 sensory features had large effects (SenSOR food, tactile, self care, smell, visual, and garment, as well as SSP sensory); 4 medium (SenSOR movement and sound, as well as SSP behavioral, registration/bystander); 4 small (SenSOR place and total, as well as SSP sensitivity/sensor and avoiding/avoider); and 1 no effect (SSP seeking/seeker). The largest sensory difference between TD and DCD subjects was for SenSOR Food (Cohen’s *d* = 1.76).

ASD and DCD subjects differed on most sensory features, but to a lesser degree than ASD and TD subjects (see Table 2). Eleven features had a large effect (maximum Cohen’s *d* = 1.46 for SSP behavioral); 2 medium, 2 small, and 1 no effect (minimum Cohen’s *d* = 0.00 for SSP sensory). Nine of 16 sensory features were selected by univariate feature selection for ASD vs. DCD decoding with a frequency greater than our 5% cutoff; of these, three (SenSOR food and tactile as well as SSP avoiding/avoider and behavioral) were selected over 90% of the time.

#### RDoC social features

Despite autism being characterized as a disorder of social interaction, many social measures did not strongly differentiate ASD from either TD or DCD (Table 3). For all paired group comparisons, the strongest differences were observed for all six SRS scores, which related to our inclusion criteria, and were therefore excluded from decoding.

Focusing on the remaining 26 social RDoC features, between ASD and TD subjects (see Table 3), 6 features that all related to social functioning had large effects (SCQ total, reciprocal social interaction, and communication, as well as CBCL total competence, social problems, and rule-breaking behavior); 3 had medium effects and related to mentalizing and alexithymia (NEPSY theory of mind verbal and communication, as well as alexithymia total); 11 had small effects and related to alexithymia, mentalizing, empathy, and affect recognition (alexithymia communication, identification, and external thinking; LOI how and why hands and face; NEPSY affect recognition; IRI personal distress and perspective taking; and EmQue prosocial motivation); and 6 relating to empathy and contextual relating of facial affect to mental state showed no to minimal effects (IRI fantasy scale, empathic concern, cognitive, and total; EmQue affective; and NEPSY theory of mind contextual).

A similar pattern with the largest effects seen for reports of social functioning and small to no effects seen for measures of social cognition was observed between TD and DCD subjects (Table 3), both of whom are *not* defined by social difficulties, and whom we do not expect to differ on social measures. Between TD and DCD subjects (see Table 3), 1 social feature outside the SRS, had a large effect (CBCL social problems); 3 related to social communication, social norms, and empathy had medium effects (SCQ total, CBCL rule-breaking; and EmQue affective); 9 related to occupational performance, alexithymia, empathy, and mentalizing had small effects (CBCL total competence; SCQ reciprocal social interaction and communication; alexithymia identification; IRI personal distress; LOI how hands and why and how face; and NEPSY theory of mind contextual); and 13 related to aspects of mentalizing, affect recognition, alexithymia, and empathy showed no to minimal effects (NEPSY affect recognition and theory of mind total and verbal; LOI how face; alexithymia communication, external thinking, and total; IRI fantasy scale, empathic concern, perspective taking, and total; and EmQue cognitive and prosocial motivation).

Between ASD and DCD subjects (whom we expected to differ on social measures), the strongest differences were once again on measures of function. Outside of the SRS (see Table 3), 3 social features, all related to occupational performance showed large effects (SCQ reciprocal social interaction and total as well as CBCL total competence); 5 related to social functioning, mentalizing, and alexithymia had medium effects (CBCL social problems, SCQ communication; NEPSY theory of mind verbal and total; and alexithymia communication); 9 related to violating social norms, alexithymia, empathy, affect recognition, and theory of mind had small effects (CBCL rule-breaking behavior; alexithymia total; EmQue affective and prosocial motivation; IRI perspective taking and empathic concern; NEPSY affect recognition and theory of mind contextual; and LOI how face); and 9 related to alexithymia, empathy, and mentalizing (alexithymia identification and external thinking; IRI personal distress, fantasy scale and total; EmQue cognitive; LOI how hands and how and why face) showed no to minimal effects.

While 20 of the 26 social measures showed some effect between ASD and TD subjects, only 7 met the 5% feature selection frequency threshold for inclusion in our ASD vs. TD decoder (Table 3): 6 of these (SCQ reciprocal social interaction, communication, and total as well as CBCL total competence rule-breaking behavior, and social problems) related to social functioning and 1 (NEPSY theory of mind total, which just met the cutoff with a selection frequency of 0.06) was a traditional measure of social cognition.

For our ASD vs. DCD decoder, only 3 social features, again, all related to social and occupational performance functioning, met the 5% threshold for inclusion in our optimized set of strongest features (Table 3): SCQ Total and Reciprocal Social Interaction as well as CBCL total competence. Very few RDoC features were selected for the optimized RDoC feature set: in addition to these three social features, only two non-social RDoC features were selected, see below. Nevertheless, supporting our feature optimization approach, performance of just these handful of measures decoded ASD from DCD better than the full set of features: decoding using the optimized set of RDoC features (Table 1) had a null *p* of 0.08 for both the MCC and BAcc performance metrics, while the full set of RDoC features performed worse with a null *p* = 0.162 for MCC and *p* = 0.183 for BAcc (Supplementary Table S1).

#### Other RDoC features

Group differences on the 16 remaining non-social RDoC features (Table 4) highlighted differences related to general functioning as measured by the CBCL and potential co-occuring conditions as well as mood and arousal.

Between ASD and TD subjects (see Table 4), 9 features had large effects (CBCL thought problems, withdrawn/depressed, anxious/depressed, aggressive behavior, and somatic complaints; Conners ADHD parent and child report; and CASI anxiety symptom count); 4 had medium effects (WASI-II VIQ; PANAS negative and positive; and PH-C total arousal); 2 had small effects (WASI-II FSIQ-2 and FSIQ-4); and 1 (WASI-II PRI) had minimal to no effects.

Between DCD and TD subjects (see Table 4), 2 features had large effects (Conners ADHD parent report and CBCL attention problem); 6 had medium effects (CBCL thought problems, withdrawn/depressed and aggressive behavior; CASI anxiety symptom count; Conners ADHD child report; and PH-C total arousal); 5 had small effects (CBCL somatic complaints; PANAS negative and positive; WASI-II FSIQ-4 and PRI); and 3 had minimal to no effects (CBCL anxious/depressed and WASI-II VIQ and FSIQ-2).

Between ASD and DCD subjects (see Table 4), 3 features had large effects (CBCL thought problems, withdrawn/depressed, and attention problems); 5 had medium effects (Conners ADHD parent report; WASI-II VIQ; and CBCL anxious/depressed, aggressive behavior, and somatic complaints); 5 had small effects (CASI anxiety symptom count; PH-C total arousal; PANAS positive; and WASI-II FSIQ-2 and PRI); and 3 had minimal to no effects (Conners ADHD child report; PANAS negative; and WASI-II FSIQ-4).

From univariate feature selection, all 9 features that showed a large effect between TD and ASD subjects, reported above, met the 5% threshold for inclusion in our ASD vs. TD decoder. Only 2 features (CBCL through problems and withdrawn/depressed) met the same criteria for our ASD vs. DCD decoder; selection frequencies were relatively low for these items at 0.3885 and 0.051, respectively.

### ASD vs. TD decoding performance

All individual and combined feature sets classified ASD from TD subjects well above chance, with *p*-values testing actual MCC performance against performance for a permuted null distribution ranging from 0.0010 (RDoC + Sensory) to 0.00020 (Sensory, Motor + Sensory, and RDoC + Motor) (Table 5).

**Table 5:** Average MCC (top) and BAcc (bottom) performance of each optimized feature set.

Feature set	ASD vs. TD MCC and BAcc (mean ± STD)	ASD vs. TD (Null *p*)	ASD vs DCD MCC and Bacc (mean ± STD)	ASD vs. DCD (Null *p*)
Motor	0.793 ± 0.156	0.00080	0.057 ± 0.183	0.190
	0.890 ± 0.081	0.0010	0.520 ± 0.070	0.190
Sensory	0.902 ± 0.110	0.00020	0.565 ± 0.207	0.0222
	0.947 ± 0.060	0.00020	0.773 ± 0.103	0.0222
M + S	0.885 ± 0.111	0.00020	0.546 ± 0.213	0.0274
	0.938 ± 0.060	0.0010	0.763 ± 0.106	0.0282
RDoC	0.889 ± 0.120	0.00040	0.377 ± 0.247	0.0832
	0.940 ± 0.064	0.00060	0.680 ± 0.119	0.0894
RDoC + M	0.949 ± 0.085	0.00020	0.383 ± 0.243	0.0881
	0.971 ± 0.049	0.00020	0.682 ± 0.117	0.104
RDoC + S	0.931 ± 0.084	0.0010	0.573 ± 0.222	0.0282
	0.961 ± 0.047	0.0010	0.777 ± 0.111	0.0276
RDoC + M + S	0.907 ± 0.091	0.00040	0.542 ± 0.224	0.0296
	0.948 ± 0.050	0.00040	0.761 ± 0.112	0.0304

Mean and standard deviation of MCC and BAcc performance across 2000 cross validation loops, as well as *p* value calculated against a null distribution reported for both decoders.

**Figure 1:**
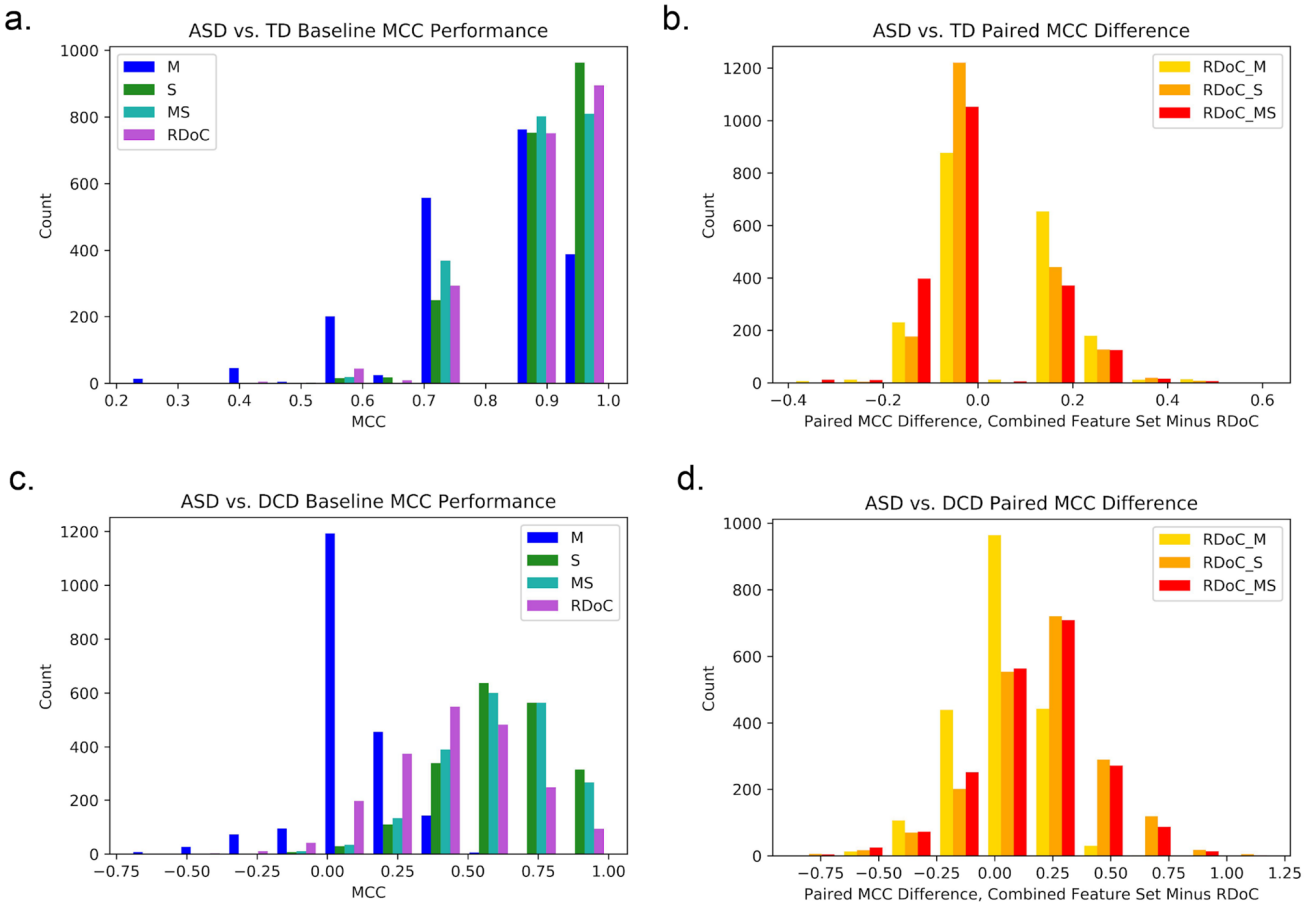
MCC decoding performance of optimized feature sets. Histogram of baseline MCC performance of separate feature sets in decoding (**a**) ASD from TD and (**c**) ASD from DCD across all 2000 CV folds in cool colors. Histogram of paired difference between combined feature sets and RDoC alone between (**b**) ASD and TD and (**d**) ASD and DCD in hot colors. *M* motor, *S* sensory, *MS* motor and sensory, *RDoC_M* RDoC and motor, *RDoC_S* RDoC and sensory, *RDoC_MS* RDoC, motor and sensory.

Motor (MCC = 0.793 ± 0.156) and sensory (MCC = 0.902 ± 0.110) measures alone performed similarly to traditional RDoC measures (MCC = 0.889 ± 0.120) in distinguishing ASD from TD subjects and had overlapping distributions (Table 5, Fig. 1a).

Combining RDoC with Motor (MCC = 0.949 ± 0.085) or Sensory (MCC = 0.931 ± 0.084) features yielded the best average classification performance (Table 5). While variance tended to be lower for combined feature sets, average performance gains were still small relative to variance for each distribution. However, inspection of the paired difference between MCC scores for each CV fold between RDoC alone and RDoC plus Motor and/or Sensory measures (Fig. 1b) show that while most folds showed no increase in performance with the addition of Motor and/or Sensory to RDoC features (the histogram peaks around zero), the distributions for each combined feature set skewed positively, indicating that when there is a difference in performance, addition of Motor and/or Sensory to RDoC features is beneficial.

### ASD vs. DCD decoding performance

Only feature sets containing sensory features decoded ASD from DCD above chance (Table 5). The Motor feature set did not discriminate between both motor-impaired groups (MCC = 0.057 ± 0.182, *p* = 0.190). Surprisingly, RDoC features alone did not perform above chance (MCC = 0.377 ± 0.247, *p* = 0.0832). In a separate analysis, inclusion of SRS scores improved RDoC performance, but as these directly related to group inclusion criteria (and are therefore clinically useful), they were not included in our SVC. Performance of any feature set containing sensory features was above chance, with the best performance observed for RDoC + Sensory features (MCC = 0.573 ± 0.222, *p* = 0.0282; this relates to a reasonable BAcc = 0.777 ± 0.111, *p* = 0.0276). Combining Sensory and both Sensory and Motor features with RDoC features improved classification relative to RDoC alone, as indicated by a shifted peak in the histogram of paired differences in performance across CV folds (Fig. 1b).

## Discussion

This study hypothesized that (1) motor and sensory features would perform similarly to RDoC features in distinguishing ASD from DCD and TD youth; and (2) that addition of motor and sensory features would boost RDoC-only classification. These hypotheses falsely presumed that RDoC features alone would successfully distinguish ASD from DCD in our sample, where ASD co-occurrence was not permitted in our DCD sample. Here we find that *only* sensory features successfully distinguished ASD from the DCD group, and aided RDoC features in the discrimination of ASD from DCD youth. Decoding ASD against the TD group, supporting H1, motor and sensory features performed similarly to RDoC features, and supporting H2, boosted RDoC performance. Together, these findings support the conclusion that sensory and motor features are important to capturing the ASD phenotype and may have clinical utility.

Classification of ASD against not just TD, but also DCD, a clinical control group whose members present motor, sensory, and even sometimes cognitive and social differences similar to those seen in ASD provides an important means of testing the practical utility of using sensory and motor features to decode ASD. Indeed, inspection of discrepancies in performance patterns between our different decoders feature sets (e.g., motor features alone did not perform well in decoding ASD against DCD) emphasizes the importance of defining a clinically relevant decoding problem when exploring the phenotypic importance of feature categories.

One clear impact of these findings is that sensory and motor features are important for describing ASD. Given this finding, it is desirable to characterize how individuals with ASD differ on these dimensions. While that specific question warrants further testing in a larger, more diverse sample with a broader range of measures, results from univariate feature selection within our sample hint at interesting preliminary differences in the ASD phenotype. Notably, while motor function, ranging from coordination to fine and gross motor skills as measured by the DCDQ and MABC checklist were highly predictive in distinguishing ASD from TD, the ability to imitate novel motor actions, as measured by the SIPT postural praxis was less predictive (Table 1). Note however that other measures of imitation ability, like the Florida Apraxia Battery, were not included here, and might provide differential results. In the sensory domain, two measures where available in this study: the SenSOR, which measures sensory over-responsivity in various domains (e.g., food, tactile, and sound) and the SSP, which measures several profiles of sensory responsiveness (e.g., avoiding, sensitivity) and behavioral regulation strategies. Here, a combination of sensory over-responsiveness in various domains (e.g., food, tactile, sound) as well most SSP profiles (Avoiding, Sensitivity, Registration, and Behavioral) were selected in the ASD vs. TD decoder 100% of the time; the SSP Sensory was selected 41.6% of the time, and the SSP Seeker only 10.6% (Table 2). Meanwhile, sensory discriminiation between ASD and DCD was more nuanced, with several features offering some discriminative utility. In our sample, the SSP Behavioral and Avoiding profiles, as well as over-responsiveness to Food and Tactile stimuli measured by the SenSOR discriminated ASD from DCD over 90% of the time, suggesting that ASD subjects specifically differed in their avoidance of certain sensory stimuli in relation to the DCD group, who also showed sensory differences relative to the TD group.

More research is needed to better understand how sensory and motor dysfunctions may be strong indicators of other disorders of social and cognitive functioning. We have externally reviewed that they are important across a range of clinical conditions^3^ and again recommend that a separate sensory domain be added to the RDoC. Our findings also endorse the recent addition of a motor domain^2^, and we look forward to the future addition of constructs that reflect everyday motor skills to this domain, which are currently not included in the RDoC motor domain. Beyond individual domains, there is room for progress at the intersection of domains. While this paper and the RDoC approach generally set up these domains as distinct, given the developmental nature of ASD as well as the interconnectedness of neural networks, it is essential to investigate functions at the intersection of domains, for example, including the role of sensation in emotional and cognitive processing and the role of sensorimotor simulation in empathy.

Our machine learning approach can be generalized beyond suggesting refinements to the RDoC. By comparing the decoding performance of combinations of disperse categories of measures, we combine results across research efforts, and can more quickly hone in on measures that are clinically meaningful to distinguish groups of interest. Furthermore, as deep-phenotyping datasets like this are collected for a larger sample of individuals falling on different parts of the ASD spectrum, we can begin to better classify subtypes of ASD^46^, which can contribute to individualized medicine. We note that our sample was taken from a current neuroimaging study on ASD, and was thus restricted to the so-called “high functioning” autism phenotype—only one of many ASD subgroups, all of which should be studied within the expanded RDoC framework to capture a deeper phenotypic characterization of ASD and its subtypes. This is an especially notable limitation with respect to the motor and sensory features, as differences in these domains are often more profound in lower functioning groups. An additional limitation was the inclusion of only right-handed participants, necessitated by the inclusion criteria of the brain imaging study from which these data were obtained; future work should include left and mixed handed individuals, who are overrepresented in ASD^47^, as handedness may relate to cognitive, sensory, and motor differences that may impact the current results. Furthermore, sex may impact the autism phenotype in the domains tested; here, our general results hold up when only males are tested (Supplementary Table S2), but this needs to be tested in a larger female sample.

Finally, it is interesting that while parent reports of social functioning (SRS, SCQ, CBCL) showed strong differences between groups, behavioral measures of social cognition (NEPSY, LOI) did not. Prior results indicate that high-functioning adolescents with ASD may deploy compensatory mechanisms to perform well on standard tests of social cognition, and seek social interaction more than their lower functioning peers, but nevertheless experience social difficulties in daily life^48^. Thus social assessments may suffer from these compensatory strategies and additional measures may be discriminative. Given the centrality of social functioning to the traditional ASD phenotype, further behavioral social measures should be investigated.

Our decoding approach can be expanded in many directions. An immediate next step addressing the aforementioned limitation would be investigating a larger sample, including individuals across the spectrum. This is especially important when considering the impact of our decoding results. For example, it is notable that motor features did not distinguish ASD from DCD in our sample. Further research is needed to determine specifically how and whether the motor profile of these groups might differ, as well as whether this finding generalizes beyond our sample: are motor difficulties consistent and equally prevalent in the general ASD population, and how does this interact with different ranges on the spectrum? Importantly does a non-motor impaired ASD subtype exist, or are motor (and sensory) differences core features of ASD?

Future studies could also investigate a broader age range (beyond the 8–17 year age range investigated here). This would reveal potential changes in the importance of motor and sensory features across the lifespan. Future studies could take advantage of pooling data across study sites, through collaborations, or through the use of research databases like the NIH’s National Database for Autism Research^49^. This approach would also allow for investigation of additional and alternative dependent measures, which might improve performance for any domain, rather than those that were available for this project. Technical issues must be addressed in the latter approach, as consolidation is difficult when data across measures within individuals is sparse or when different measures are used by different research groups to measure similar constructs. However, as larger datasets are accreted, the clustering and individualized psychiatry endeavors suggested above can advance. Additionally, measures related to the restricted, repetitive, and stereotyped behaviors that are considered characteristic of ASD were not explored in this study as these behaviors may have several explanatory causes and any precise mapping to domains is unclear. Relating symptom-level research in restricted, repetitive, and stereotyped behavior to RDoC, motor, and sensory domains is an area of future study. Furthermore, another future step would be to circumvent the limitations of self-report data. This could be done by collecting objective data from psychometrics, psychophysiology, brain imaging, genetics, and potentially gut microbiota composition. Finally, it would be important to conduct a similar study using other pertinent clinical control groups beyond the DCD group used here.

The long term goal following from this research would be to develop comprehensive, yet succinct clinical assessment batteries for the differential diagnosis of ASD against other clinical disorders, as well as for subtyping within ASD. Our findings preliminarily indicate that sensory and motor measures may play a prominent role in such batteries, and may better inform individualized treatment.

## Methods

### Subjects

Study participants were recruited as part of an ongoing neuroimaging study (NIH Award No. R01HD079432) and included 32 youth with ASD; 26 with DCD; and 34 TD controls, all matched for age, biological sex, and full scale IQ (Table 6). Of these, two ASD and one TD subject were excluded for missing data (see below).

**Table 6:** TD, ASD, and DCD subject groups.

Subject group	*N*	Age in years (mean ± STD)	FSIQ-IV (mean ± STD)	VCI (Mean ± STD)	PRI (mean ± STD)
TD	33 (11 female)	11.9 ± 2.3	113.6 ± 11.0	114.7 ± 11.1	109.2 ± 13.2
ASD	30 (7 female)	12.1 ± 2.2	108.0 ± 18.8	105.4 ± 17.1	109.6 ± 21.0
DCD	26 (11 female)	11.8 ± 2.3	110.2 ± 17.1	114.0 ± 15.6	104.0 ± 20.1
ANOVA (F, p)	–	0.27, 0.77	1.00, 0.37	3.71, 0.028	0.82, 0.44

Subject groups were matched for age, and IQ; F-statistic and p-value are reported for one-way ANOVAs comparing the three group means for each measure.

All subjects were required to (1) be between 8 and 17 years of age; (2) be in mainstream education, performing at or near grade level and have a WASI^50^ full scale IQ (FSIQ-IV) score greater than 80; (3) be right-handed; (4) be fluent in and have a parent fluent in English; (5) not have a history of loss of consciousness for greater than 5 min; (6) have a gestational age greater than 36 weeks; and (7) be positively screened for MRI compatibility. Three subjects (two ASD and one DCD) did not meet the full scale IQ cutoff, but were included as either their perceptual reasoning index (PRI) or verbal comprehension index (VCI) scores were greater than 80. Additionally, TD subjects (1) could not have any concern of ASD, including a maximum SRS^51^ t-score of 59; (2) or parents or siblings with ASD; (3) or any psychological diagnosis or neurological disorder; or (4) score at or below the twenty-fifth percentile on the MABC^52^.

In addition to the general inclusion criteria, ASD subjects needed to have both (1) a community diagnosis of autism and (2) meet criteria for an ASD diagnosis according to the Autism Diagnostic Observation Schedule-Second Edition^53^ (ADOS-2) and/or the Autism Diagnostic Interview, Revised^54^ (ADI-R). Two of our female ASD subjects did not meet criteria on the ADOS-2, but did meet on the ADI-R and had received a clear diagnosis earlier in life and had received substantial intervention. Further ASD inclusion criteria included: (3) subjects could not have an additional diagnosis of a neurological or psychological disorder, with the exception of attention deficit disorder, or a clinically-managed generalized anxiety or depressive disorder, both of which commonly co-occur with ASD; (4) the dose of any current psychotropic medications must be stable. In the ASD group, ten individuals were taking previously prescribed psychotropic medications for ADHD, and five individuals were taking psychotropic medication for depression or anxiety.

In addition to the above general inclusion criteria, the primary criteria for inclusion in the DCD group was receiving a score at or below the 16th percentile on the MABC-2. A diagnosis of DCD is not commonly given in California where the study was conducted, and therefore was not required. Therefore, these subjects should be considered to be probable DCD, but for the sake of readability are called DCD in this manuscript. DCD subjects could not have a first-degree relative with ASD, nor an additional diagnosis of a neurological or psychological disorder, with the exception of attention deficit disorder, or a clinically-managed generalized anxiety or depressive disorder. In the DCD group, five individuals were taking previously prescribed psychotropic medications at the time of the study, four of which were for ADHD, and one of which was for both ADHD and anxiety. Children who scored in a range of 65–74 on the SRS-2, indicating potential social differences, were administered the ADOS-2 assessment and excluded from the study if they met criteria for ASD.

All participants gave written informed assent (consent form for minors) and had written informed parental permission before the research began. They received monetary compensation for their participation at a rate of $15 per hour. The study protocol was approved by the University of Southern California Institutional Review Board in accordance with the Declaration of Helsinki.

### Measures

Behavioral assessments, self-report, and parent-report measures were collected. All behavioral assessments were administered by trained research staff. All item scores were double-entered and verified using a REDCap (Research Electronic Data Capture) database^55,56^.

### Design

All available measures were mapped onto either (1) the original RDoC domains, (2) a motor domain, or (3) a sensory domain (Table 7). Measures related to group inclusion criteria (i.e., MABC and SRS) were not used. Following domain mapping, feature sets from each category (RDoC, sensory, and motor), and combinations thereof were used in both an ASD vs. TD and an ASD vs. DCD decoder.

**Table 7:** Mapping of available measures to RDoC, motor, and sensory domains.

Domain	Measures
**Original RdoC (42 features)**	
Negative valence systems	1. Positive and Negative Affect Scale for Children^65^ (PANAS-C; negative affect) 2. Childhood Anxiety Sensitivity Index^66^ (CASI)(anxiety symptom count) 3. Child Behavior Checklist^67^ (CBCL)(anxiety, withdrawn and aggression subscores)
Positive valence systems	1. PANAS-C (positive affect)
Cognitive systems	1. Wechsler Abbreviated Scale of Intelligence, 2nd Edition^50^ (WASI-II) (IQ scores) 2. Conners^68^ (ADHD symptoms) 3. CBCL (thought problems and attention subscores)
Social processes	1. NEPSY-II^69^ (affect recognition and theory of mind subscores) 2. Interpersonal Reactivity Index^70^ (IRI) 3. Empathy Questionnaire^71^ (EmQue) 4. Alexithymia^72^ (total and subscores) 5. Level of Inference task^73^ (LOI) (why/how face/hand judgments) 6. CBCL (social problems, competence^a^, and rule-breaking subscores) 7. SCQ^74^ (total and subscores excluding RRB)
Arousal and regulatory systems	1. Physiological Hyperarousal Scale for Children^65^ (PH-C) 2. CBCL (somatic subscore)
Motor (7 features)	1. MABC-Checklist^52^ (total and subscores)^b^ 2. The Developmental Coordination Disorder Questionnaire^75^ (DCDQ) (total and subscores) 3. Sensory Integration and Praxis Test^76,77^ (Postural Praxis)
Sensory (16 features)	1. Sensory Over-Responsivity^78^ (SenSOR)(total and subscores) 2. Short Sensory Profile 2^79^ (SSP)(total and subscores)

^a^The CBCL competence score reflects participation and quality of performance in activities, social settings, and school. While it reflects aspects of cognitive performance, we include it in the social category as a measure of functionality in typical social environments.

^b^The MABC and MABC checklist are normed for children up to 17 years of age. Our sample included one 17 year old, who was scored according to the norms used for a child aged 16 years and 11 months.

### Analysis

All analyses were performed using custom software written in Python.

#### Dataset characterization

To explore how the groups differed on each feature and feature category, Cohen’s *d* effect sizes are reported for each feature for each paired group comparison in tables organized by feature category. These tables are sorted by the importance of each feature in decoding ASD according to their univariate feature selection frequency counts (see below). To confirm that sensory and motor features are non-redundant with RDoC features, Spearman correlations were calculated between RDoC and sensory and between RDoC and motor features.

#### Decoding

**Figure 2:**
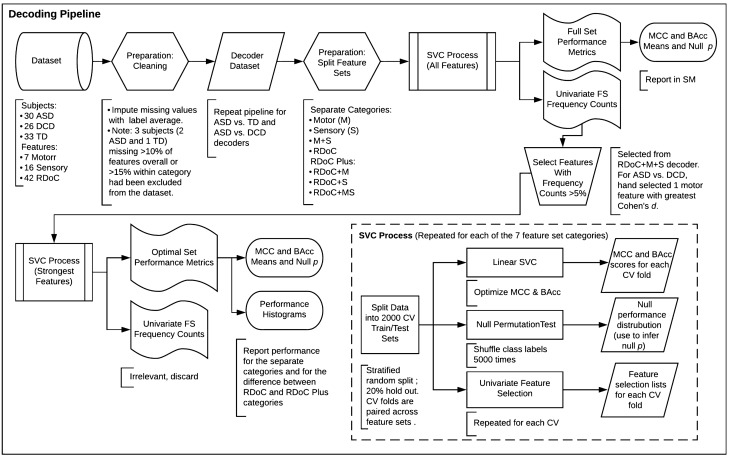
Decoding pipeline.

Individuals were classified by diagnosis in both an ASD vs. TD and ASD vs. DCD decoder using linear support vector classifiers (SVCs). To compare the performance of RDoC, Motor, and Sensory feature sets in decoding ASD, features in our dataset from each category separately, as well as in combination, were subjected to our SVC process, detailed below. The scikit-learn^57^ Python package was used for decoding. Figure 2 illustrates the entire decoding pipeline.

##### Data preparation

Subjects missing more than 10% of features overall or 15% from any single category were excluded from analysis. Missing data in the remaining subjects was imputed using the mean value of the subject’s assigned group (ASD, DCD, TD) for that feature. Following the imputation of missing values, all raw scores were z-transformed within each CV fold (see below).

##### Cross validation

For each decoder (ASD vs. TD and ASD vs. DCD), the data was randomly split into 2000 cross validation train/test sets (at a rate of 80%/20%) using stratified sampling such that each target class was proportionally represented in each split. Code was written to confirm that all 2000 splits were unique and independent. To prevent data leakage with normalization, in each fold, each feature in the training set was z-scored, and the training parameters were used to scale the test set.

For reproducibility purposes, and so that CV splits were paired across feature sets for later comparison, all random number generators were fixed at the beginning of the script and CV folds were initialized prior to any training. To ensure that the results were not confounded by this fixed initialization, the entire analysis pipeline was repeated 10 times with random initialization. The results were essentially the same across 10 repeats.

##### Performance metrics

Classification performance was assessed with Matthew’s Correlation Coefficient (MCC) and Balanced Accuracy (BAcc). MCC is recommended for class imbalanced decoding problems where both classification successes (e.g., true positives) and classification errors (e.g., false positives) must be considered^58^. A benefit of the MCC metric is that it is derived from all four elements of the classification confusion matrix, and therefore provides a comprehensive representation of decoding performance in a single number. MCC scores were calculated by scikit-learn, following the standard definition^59^, and ranged from − 1 to 1, where a score of 1 represents perfect prediction (i.e., all ASD subjects classified as ASD), a score of − 1 represents inverse prediction (e.g., all ASD subjects classified as DCD subjects in the ASD vs. DCD decoder), and a score of 0 represents random prediction. BAcc scores are accuracy scores adjusted for class imbalance. Because of their comprehensive reporting of performance, MCC scores are discussed in the manuscript and reported in Fig. 1, our sole results figure. BAcc scores are reported alongside MCC results in Table 5, which reports decoding performance of optimized feature sets, but are otherwise only reported in Supplemental Materials for readers who find accuracy scores more intuitive than MCC.

##### Feature selection

Univariate feature selection was used to investigate the relative importance of each feature in distinguishing ASD from either TD or DCD. Feature selection was performed rather than a feature reduction technique like PCA in order to relate decoding findings directly back to assessments that might be used in clinical practice.

Multivariate feature selection approaches (such as forward selection, backward selection, and recursive feature elimination) have been frequently used in the literature to select explanatory features. However, a growing body of research in statistics has suggested that these approaches might lead to overfitting to data, yield false confidence intervals, generate too low of p values, and confuse actual predictor features with noise features^60,61^. To circumvent these issues, a feature selection approach based on univariate correlation and cross-validation has been suggested and used in neuroscience studies, where the features are intrinsically highly collinear^62,63^. Based on the arguments and findings of those previous studies, this latter approach was used in the current study.

Univariate feature selection was performed for each of the initial 2000 CV folds, and the frequency count—the total number of times a feature was selected across CV iterations, divided by the total number of iterations, 2000—was saved. This process was performed for each individual (RDoC, Motor, and Sensory) and combined (e.g., RDoC + Motor + Sensory) feature set, as well as for each decoder (ASD vs. TD and ASD vs. DCD).

Frequency counts from the separate feature categories (e.g., Motor alone) were similar to those from the full RDoC plus Motor and Sensory feature set, and the latter frequency counts were reported as part of the dataset characterization described above.

Additionally, any feature that was selected in more than 5% of CV iterations was selected to train our final SVC. In the ASD vs. DCD decoder, no motor features were selected by univariate feature selection as MCC scores for the motor feature set did not exceed chance performance. In order to be able to test a final SVC process using optimized features, a single motor feature—DCDQ Motor Planning—was hand selected for the ASD vs. DCD decoder because it had the greatest difference between the two groups (Cohen’s *d* =  − 0.29).

Because our feature optimization routine was based on a summary of many univariate feature selection results and independent of any particular training set, it is non-circular and permissible. For comparison purposes, decoding results for the full feature sets are reported in the Supplementary Materials.

##### Control: age testing

To control for the effects of age interacting with features to predict group membership, in a separate analysis, age was included as a feature for each feature set. As the addition of an age feature did not change the overall results, and because it was also never selected in univariate feature selection, it was not considered as a variable of interest in our final models. Additionally, as described above in the Subjects section, all three groups were matched for age.

##### Statistical testing

Permutation testing whereby class labels were shuffled 5000 times was used to test decoding performance for each feature set for each decoder against chance performance and obtain *p*-values indicating the statistical significance of performance against a null distribution.

##### Model comparison

Following our aim to compare the classification of ASD using RDoC and sensorimotor features, we compared the mean classification performance across all 2000 CV folds of each individual and combined feature set. Because performance distributions were non-parametric and skewed towards ceiling performance, we chose to report histograms to allow readers to visually inspect the relative performance of (1) each feature set category separately (RDoC, M, S, and MS) as well as (2) the added benefit of including other features with RDoC features, derived from paired comparisons across CV folds (e.g., RDoC + M + S minus paired performance from RDoC alone).

## Supplementary Information

Supplementary Information.

## Data Availability

The raw data supporting these findings have been deposited on the Open Science Framework (https://doi.org/10.17605/OSF.IO/UENM9)^64^.

## Code availability

The core custom Python code supporting these findings have been deposited on the Open Science Framework (https://doi.org/10.17605/OSF.IO/UENM9)^64^.
