# Supplementary material for: Motor and sensory features successfully decode autism spectrum disorder and combine with the original RDoC framework to boost diagnostic classification
Source: Sci Rep. 2021 Apr 9;11:7839. doi: 10.1038/s41598-021-87455-w (PMC8035204; doi:10.1038/s41598-021-87455-w)
Supplement: Supplementary file 1 — Supplementary Information. [file 41598_2021_87455_MOESM1_ESM.pdf]

Motor and Sensory Features Successfully Decode Autism Spectrum Disorder and Combine with the Original RDoC Framework to Boost Diagnostic Classification

Laura A. Harrison[1,2*], Anastasiya Kats[2], Emily Kilroy[1,2], Christiana Butera[1,2], Aditya Jayashankar[1,2], Umit Keles[3] and Lisa Aziz-Zadeh[1,2]

University of Southern California

Author Note

[1]USC Chan Division of Occupational Science and Occupational Therapy, University of Southern California, Los Angeles, CA, USA

[2]Brain and Creativity Institute, University of Southern California, Los Angeles, CA, USA

[3]Division of Humanities and Social Sciences, California Institute of Technology, Pasadena, CA, USA

Funding statement. This research was supported by the NIH Award No. R01HD079432.

Acknowledgments: We would like to thank the research participants and their families who make this research possible. We would also like to thank Jonas Kaplan, Morteza Dehghani, and Kingson Man for consulting on machine learning techniques; Sharon Cermak, Marian Williams, and Emily Harnin for assisting with clinical diagnosis; and Mirella Dapretto, Susan Bookheimer, and Ralph Adolphs for discussions related to this topic. We would also like to thank past and present OTD residents in our laboratory — Alyssa Concha, Cristin Zeisler, Priscilla Ring, Ryann MacMurdo, Alexis Nalbach, and Anusha Hossain — as well as our undergraduate research assistants who assisted with data collection and entry.

Correspondence concerning this article should be addressed to Laura Harrison.

Contact: laurah@usc.edu

Supplementary Materials

Supplementary Table 1. Average MCC (top) and BAcc (bottom) performance of each full feature set. Mean and standard deviation of MCC and BAcc performance across 2000 cross validation loops, as well as *p* value calculated against a null distribution reported for both decoders.

**Feature Set**	**ASD vs. TD MCC and BAcc (Mean±STD)**	**ASD vs. TD (Null *p*)**	**ASD vs DCD MCC and BAcc (Mean±STD)**	**ASD vs. DCD (Null *p*)**
Motor	0.793±0.156	0.00080	-0.040±0.285	0.591
	0.890±0.081	0.0010	0.479±0.123	0.591
Sensory	0.902±0.110	0.00020	0.628±0.208	0.0136
	0.947±0.060	0.00020	0.805±0.105	0.0170
M+S	0.885±0.111	0.00020	0.531±0.212	0.0250
	0.938±0.060	0.0010	0.756±0.105	0.0372
RDoC	0.863±0.130	0.00020	0.328±0.256	0.162
	0.926±0.069	0.0010	0.655±0.123	0.183
RDoC + M	0.887±0.107	0.00040	0.302±0.249	0.175
	0.934±0.063	0.00060	0.643±0.119	0.175
RDoC + S	0.911±0.098	0.00020	0.492±0.236	0.0568
	0.950±0.055	0.00020	0.736±0.116	0.0660
RDoC + M + S	0.930±0.088	0.00020	0.479±0.239	0.0640
	0.960±0.051	0.00020	0.730±0.118	0.0652

Supplementary Table 2. Average MCC (top) and BAcc (bottom) performance of each optimized feature set for male subjects only. Mean and standard deviation of MCC and BAcc performance across 2000 cross validation loops, as well as *p* value calculated against a null distribution reported for both decoders.

Feature Set	ASD vs. TD MCC and BAcc (Mean±STD)	ASD vs. TD (Null *p*)	ASD vs DCD MCC and BAcc (Mean±STD)	ASD vs. DCD (Null *p*)
Motor	0.852±0.122	0.00080	-0.036±0.333	0.581
	0.918±0.068	0.0012	0.483±0.152	0.577
Sensory	0.961±0.077	0.00020	0.585±0.298	0.0463
	0.978±0.042	0.00020	0.777±0.149	0.0696
M+S	0.929±0.098	0.0010	0.594±0.292	0.0494
	0.961±0.054	0.0010	0.782±0.147	0.0756
RDoC	0.985±0.056	0.00160	0.566±0.269	0.0743
	0.992±0.031	0.00160	0.767±0.133	0.0724
RDoC + M	0.988±0.053	0.00120	0.557±0.269	0.0742
	0.993±0.030	0.00120	0.762±0.133	0.0742
RDoC + S	0.963±0.076	0.00080	0.555±0.277	0.0786
	0.980±0.042	0.00080	0.766±0.139	0.0768
RDoC + M + S	0.962±0.074	0.0010	0.555±0.275	0.0758
	0.979±0.041	0.0010	0.767±0.138	0.0780

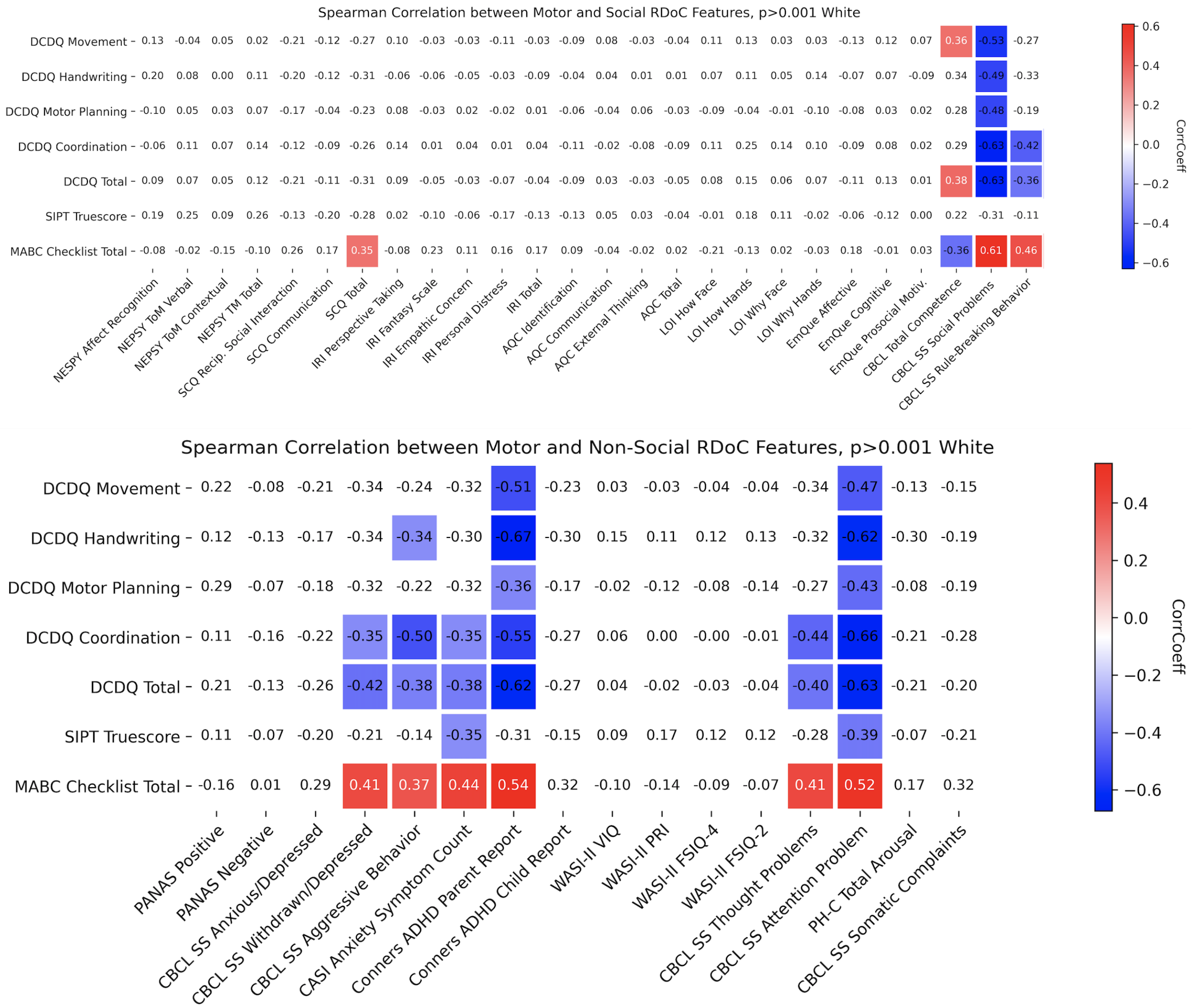

Supplementary Figure 1. Spearman correlation of RDoC and Motor features. In concordance with Tables 3 and 4, RDoC features are separated into social (top) and non-social (bottom) constructs. The strength of correlations where $p<0.001$, uncorrected, are displayed with heatmap coloring; other cells are white.

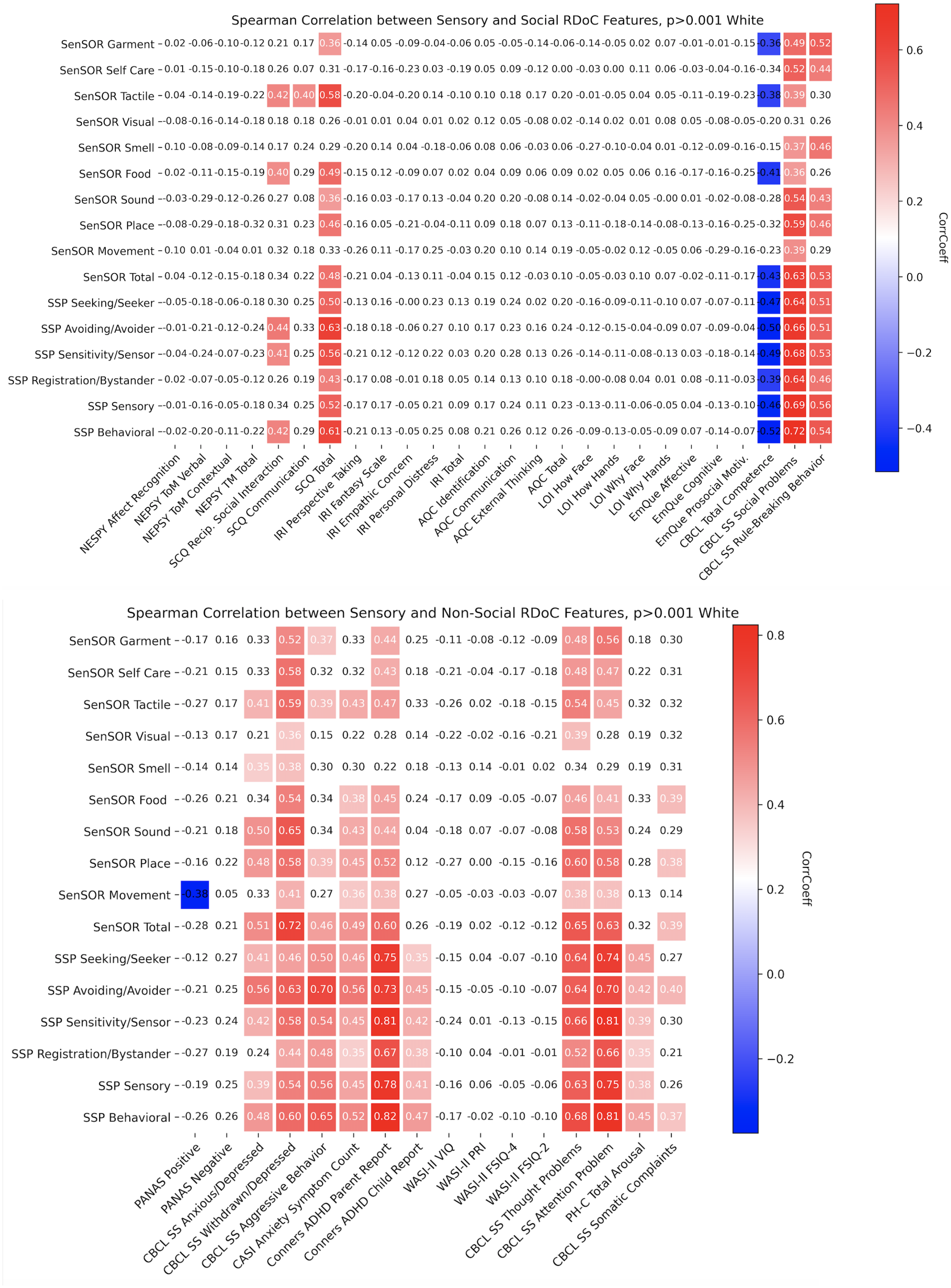

Supplementary Figure 2. Spearman correlation of RDoC and Sensory features. In concordance with Tables 3 and 4, RDoC features are separated into social (top) and non-social (bottom) constructs. The strength of correlations where p<0.001, uncorrected, are displayed with heatmap coloring; other cells are white.

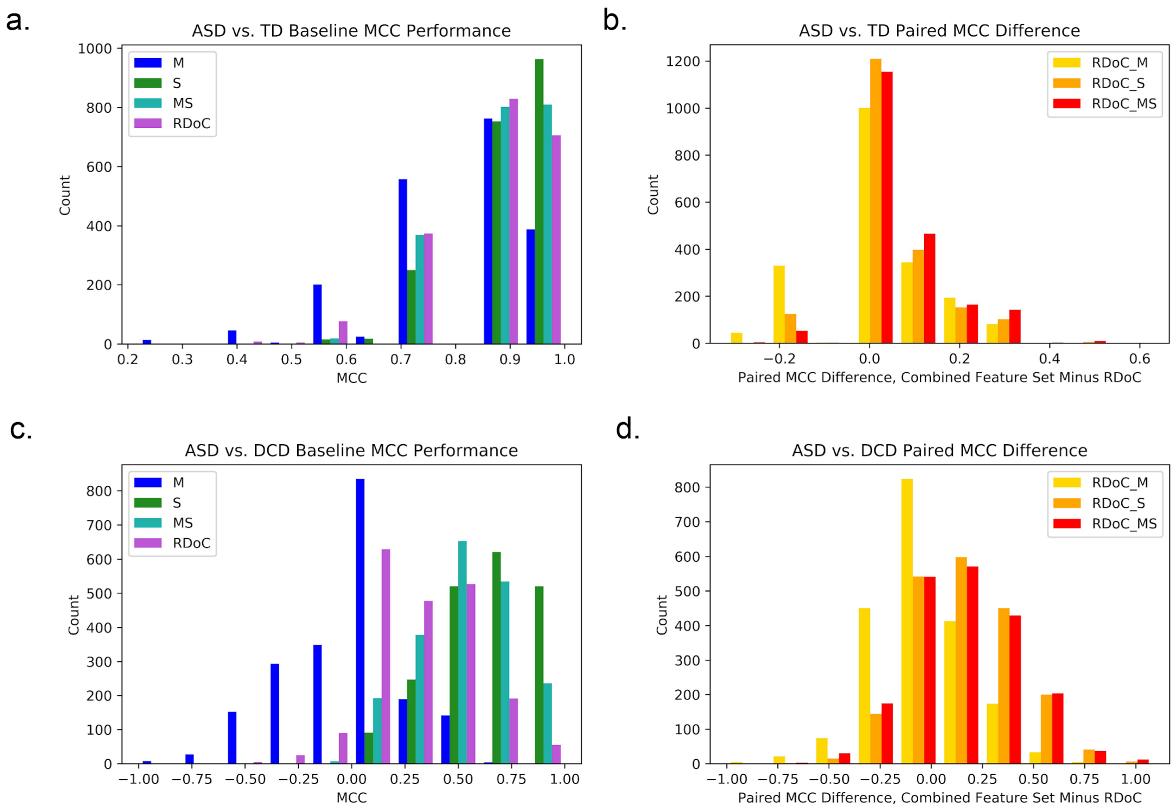

Supplementary Figure 3. MCC Decoding Performance of Full Feature Sets. Same conventions as Figure 1.

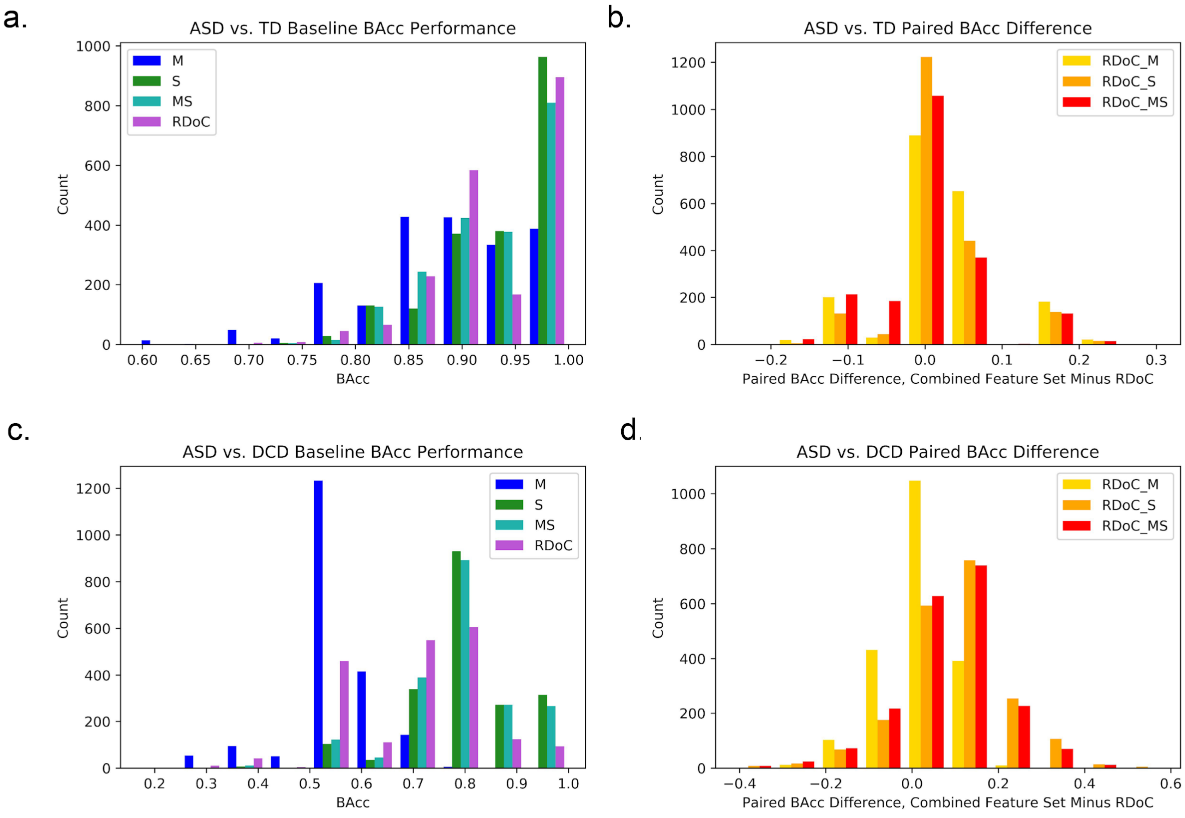

Supplementary Figure 4. BAcc Decoding Performance of Optimized Feature Sets. Same conventions as Figure 1.

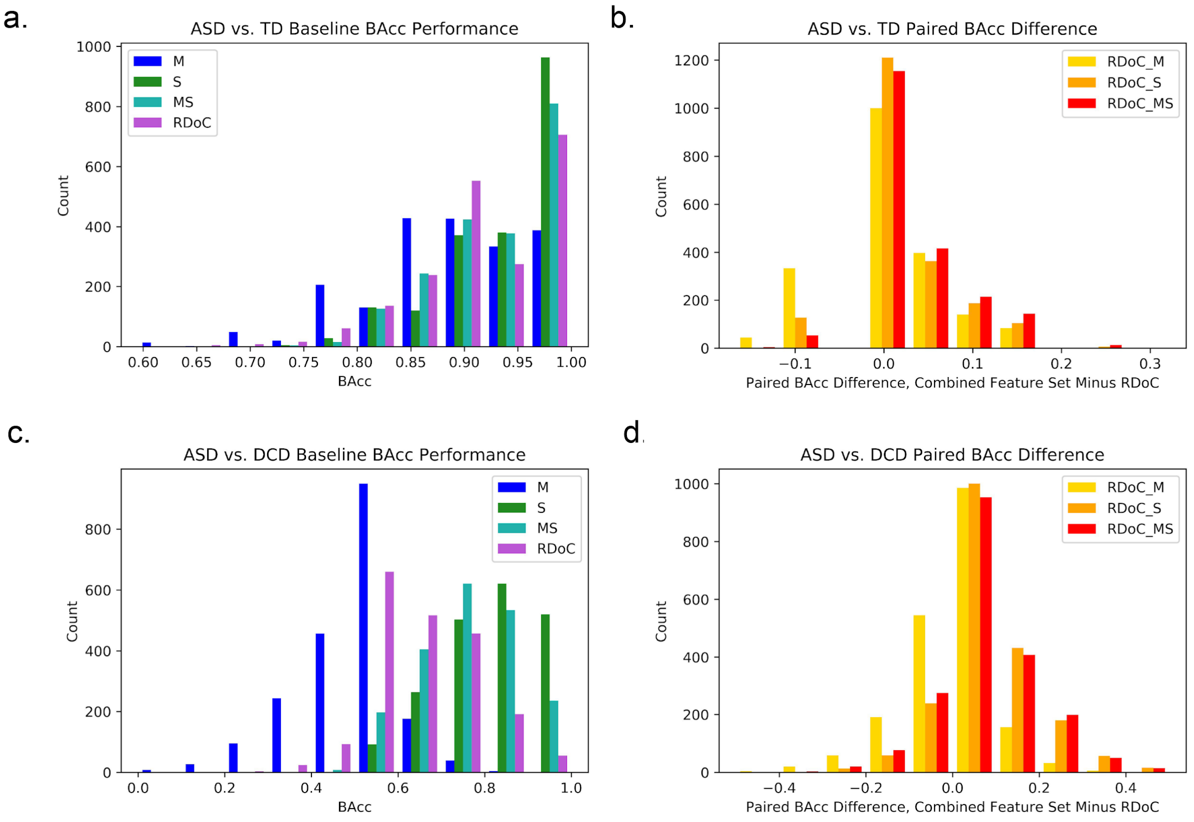

Supplementary Figure 5. BAcc Decoding Performance of Full Feature Sets. Same conventions as Figure 1.
